# Supplementary material for: Thermo-Responsive ZnPc-g-TiO2-g-PNIPAM Photocatalysts Sensitized with Phthalocyanines for Water Purification under Visible Light
Source: Molecules. 2022 May 22;27(10):3330. doi: 10.3390/molecules27103330 (PMC9143362; doi:10.3390/molecules27103330)
Supplement: Supplementary file 1 [file molecules-27-03330-s001.zip › molecules-1722366-supplementary.pdf]

Supporting Information

Thermo-responsive ZnPc-*g*-TiO_2-*g*-PNIPAM photocatalysts sensitized with phthalocyanines for water purification under visible light

Synthesis of 2, 9(10), 16(17), 23(24)-tetrakis[(3-carboxyacrylamide) phthaloc y-aninato] zinc (ZnPc)

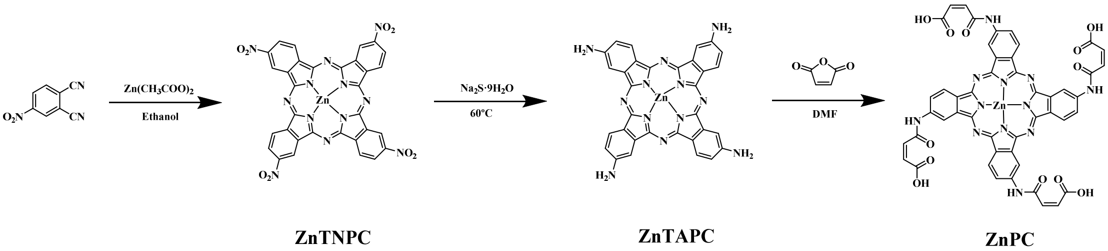

Scheme S1. The procedure of ZnPc

Tetraamino phthalocyanine zinc was prepared according to our previous study [1]. Firstly, 1 mmol tetraamino phthalocyanine zinc and 4 mmol maleic anhydride were dissolved in 30 mL *N, N*-dimethylformamide and maintained at 50°C for 3 h. Secondly, 200 ml deionized water was added into the solution and products were separated by centrifugation. Thirdly, the products were dissolved in 0.1 mol/L NaOH solution and filtered. After filtration, the product was dissolved into deionized water (pH=2). Finally, products were washed with ethanol and water, and then dried at 60 °C in a vacuum, yield 67%. ^{1}H-NMR (DMSO-d_6, ppm): 11.28 (d, 4H), 9.81 (d, 4H), 9.32 (s, 4H), 8.46 (d, 4H), 6.78 (d, 4H), 6.53 (d, 4H), 6.26 (s, 4H).

Photocatalytic experiments

The RhB and MB photodegradation by TiO_2 and ZnPc-*g*-TiO_2-*g*-PNIPAM were carried out by using a photocatalytic device (PLS-SXE300). The light source was a Xe lamp (300 W) equipped with visible area bandpass filter (λ > 420 nm). Eight milligrams of the photocatalyst was suspended in the RhB aqueous solution (40 mL, 1.0×10^{-5} M) or the MB aqueous solution (40 mL, 1.0×10^{-5} M). Before the degradation experiments, the suspensions were stirred for 60 min in the dark to reach the equilibrium of adsorption-desorption. H_2O_2 (2 mL) was used as a control. Then, the suspensions were irradiated under the xenon lamp. ZnPc-*g*-TiO_2-*g*-PNIPAM was separated with high-speed centrifugation at 45 °C and TiO_2 was separated at 25 °C. The degradation concentration of RhB and MB aqueous solution was analyzed by UV-vis 2600 spectrophotometer.

Analysis of reactive species

The formation of hydroxyl radicals ($OH^{\cdot}$) and superoxide radicals ($O_2^{\cdot-}$) generated by ZnPc-*g*-TiO_2-*g*-PNIPAM under visible light irradiation was detected by various scavengers. IPA (6.5 mM) and BQ (0.5 mM) were employed as scavengers to investigate $OH^{\cdot}$ and $O_2^{\cdot-}$, respectively. IPA and BQ were added to the RhB solution after the adsorption–desorption equilibrium of the photocatalyst. The analysis method was same to the photocatalytic experiment.

Detection limits

The detection limits (DL) of major pollutants were tested. According to the Technical Guidelines for Development and Revision of National Environmental Monitoring Methods and Standards (HJ168-2010), DL was calculated on the basis of $t_{(n-1, p)} \times S$, where S is the standard deviation of ten blank samples, n is the number of blank samples and p is probability (p=0.99). The $t_{(9, 0.99)}$ value was 2.82 in this experiment. The DLs of RhB, MB and TC•HCl were 0.07 mg/L, 0.16 mg/L and 0.03 mg/L, respectively. The rationality of each result was tested, and it was proved that the data were reliable.

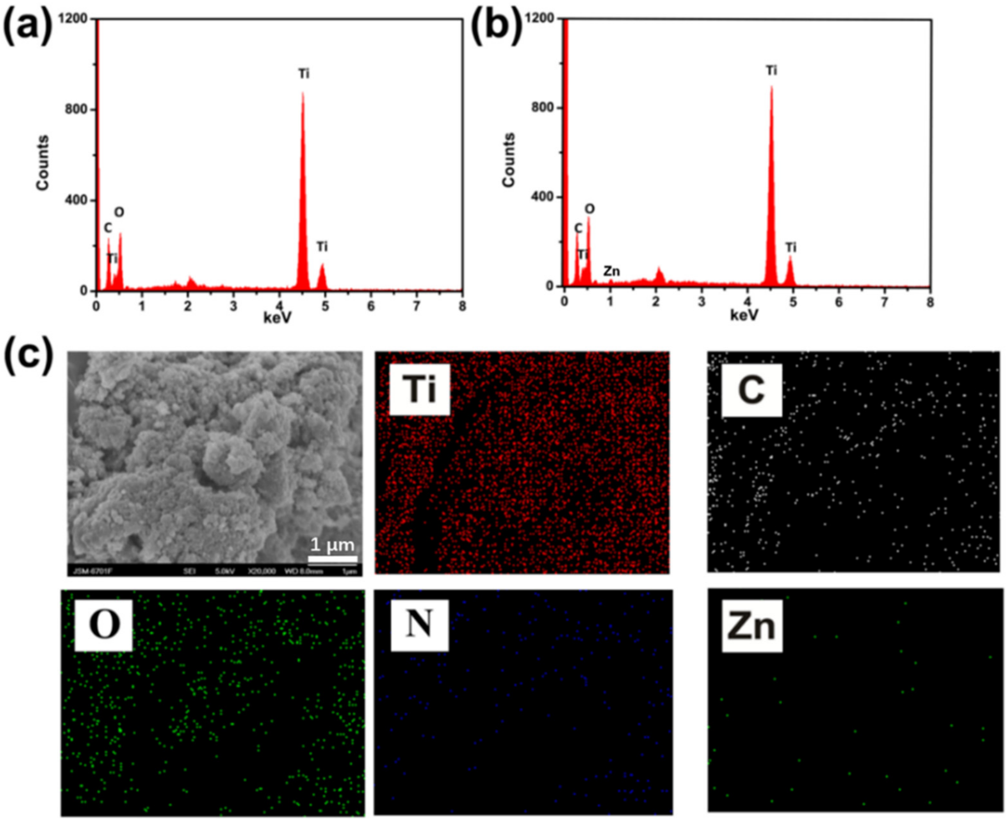

Figure S1. EDS spectrum of TiO_2-*g*-PNIPAM (A) and ZnPc-*g*-TiO_2-*g*-PNIPAM (B), the elemental mapping images of ZnPc-*g*-TiO_2-*g*-PNIPAM (C).

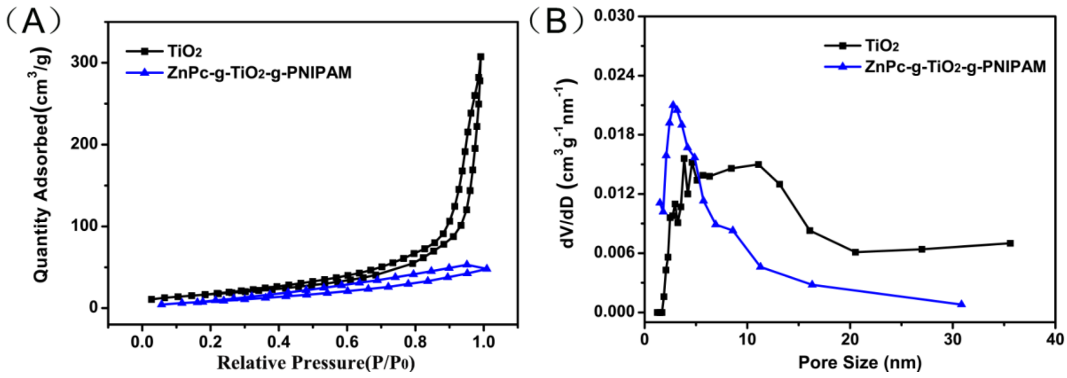

Figure S2. N_2 sorption isotherms (A) and pore-size distribution (B) of TiO_2 and ZnPc-*g*-TiO_2-*g*-PNIPAM.

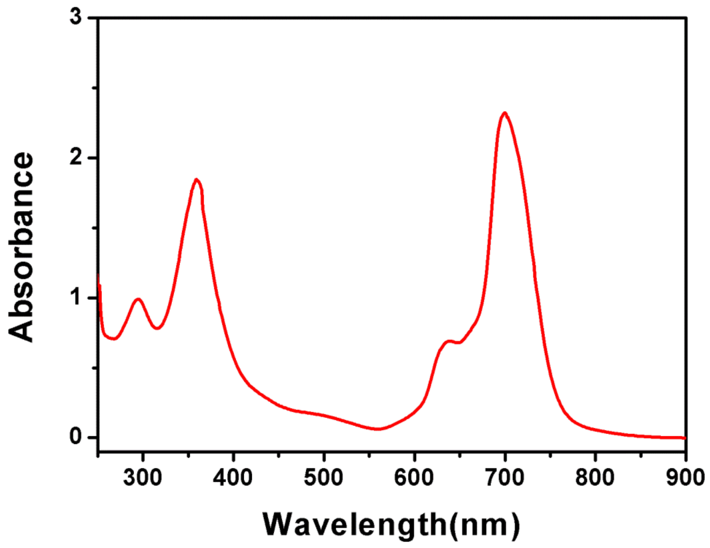

Figure. S3. UV-vis spectra of ZnPc (0.5 mg/mL) in dimethyl sulfoxide

confidence interval

The confidence interval was calculated on the basis of $\bar{x} \pm t\frac{s}{\sqrt{n}}$, where s is the standard deviation, $\overline{X}$ is the sample mean, n is the number of experiments (n=5), the confidence level is 95 % and t is 2.776. The confidence interval of TiO_2 and ZnPc-*g*-TiO_2-*g*-PNIPAM bandgaps were 3.17-3.19 and 2.92-2.93.

Critical solution temperature measurement

The lower critical solution temperature (LCST) of ZnPc-*g*-TiO_2-*g*-PNIPAM (0.1 mg/mL) in aqueous solution was measured by turbidimetry with the UV-2600 spectrophotometer. The change in transmittance at 500 nm was monitored by heating the solution at a rate of 1 °C/min. We chose the temperature corresponding to a 10% decrease in the maximum transmittance as the LCST of the sample.

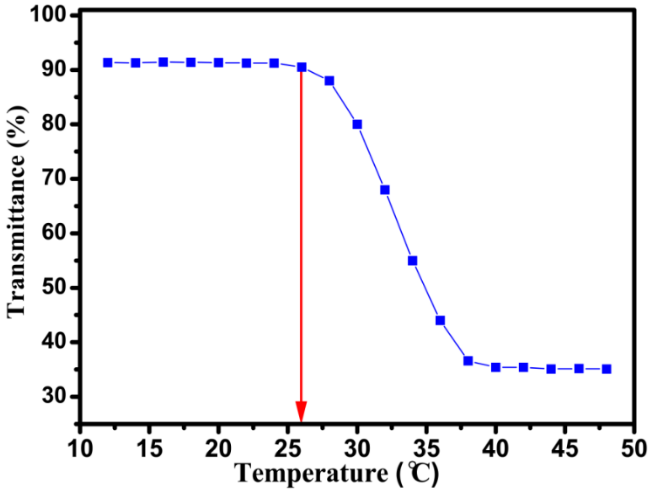

Figure S4. ZnPc-*g*-TiO_2-*g*-PNIPAM (0.1 mg/mL) transmittance with temperature in water (λ=500nm)

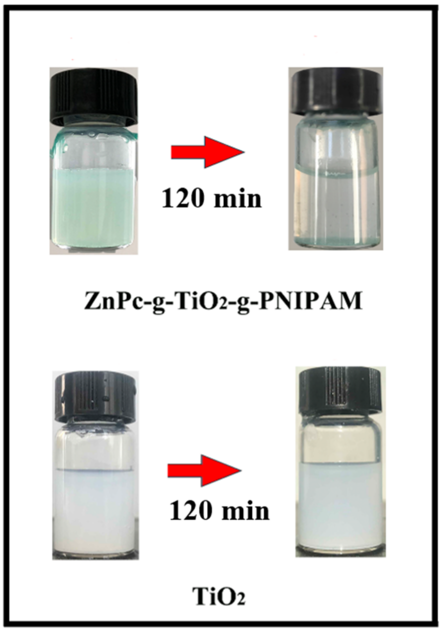

Figure S5. TiO_2 (0.5 mg/mL) and ZnPc-*g*-TiO_2-*g*-PNIPAM (0.5 mg/mL) dispersed in aqueous solution were left to rest for 120 min at 45°C.

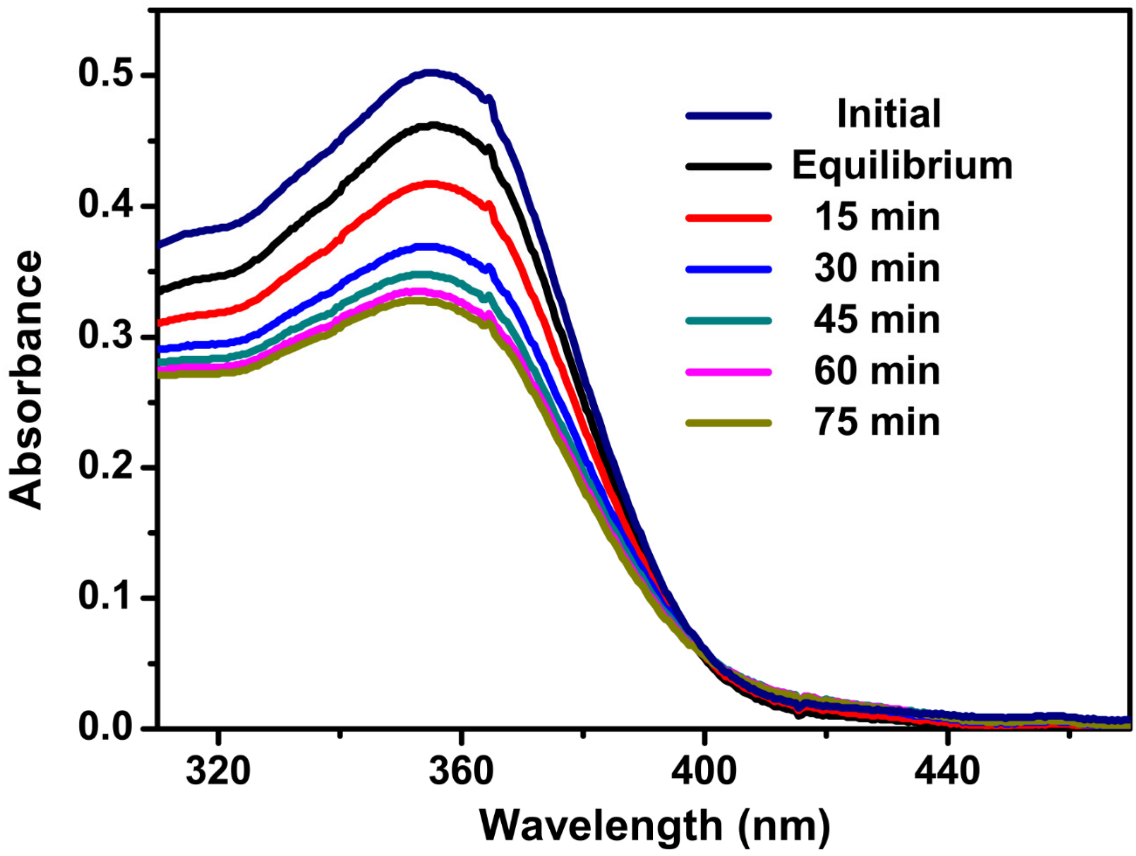

Figure S6. The removal of TC•HCl (10 mg/L) with ZnPc-*g*-TiO_2-*g*-PNIPAM/H_2O_2 (0.3 g/L) under visible light.

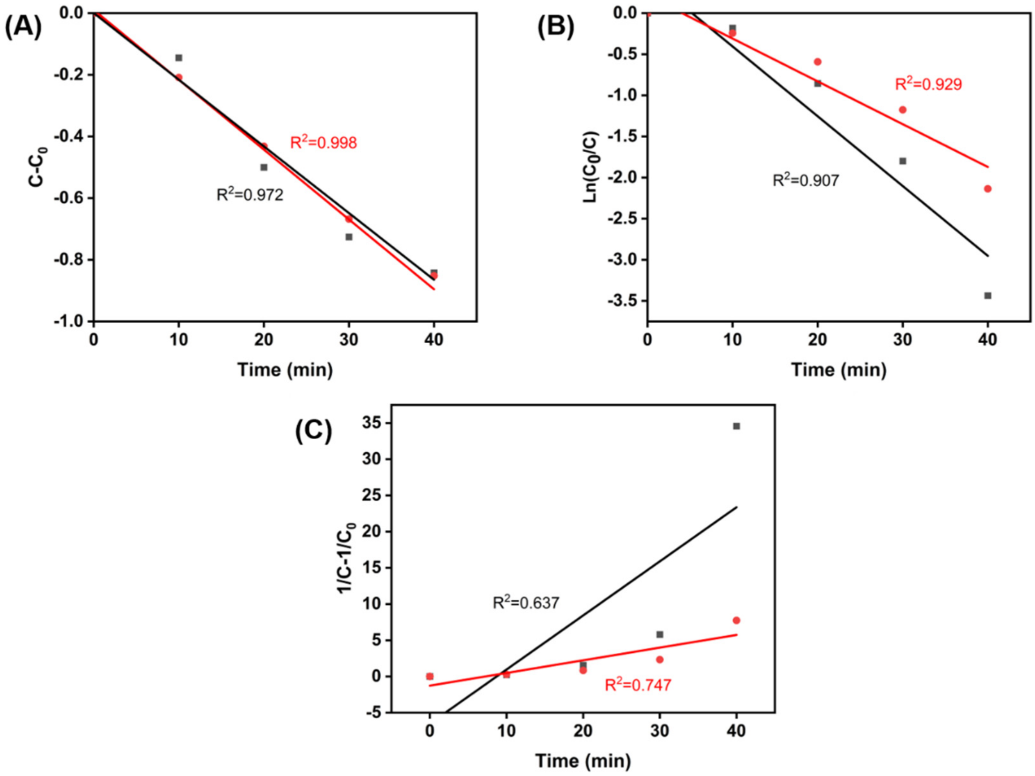

Figure S7. Kinetics curves of RhB (red line) and MB (black line) degradation at 40 mL, 1.0 × 10^{-5} M of the dye solution, the optimum amount of catalyst (300 mg/L), and with 20 mL H_2O_2.

Reference

[1] Li Y, Zhao D, Li Y, Liu Y, Duan Q, Kakuchi T Synthesis of water-soluble and thermoresponsive phthalocyanine ended block copolymers as potential photosensitizer. Dyes Pigments **2017**, *142*, 88-99.
